# Supplementary material for: VirB, a transcriptional activator of virulence in Shigella flexneri, uses CTP as a cofactor
Source: Commun Biol. 2023 Nov 25;6:1204. doi: 10.1038/s42003-023-05590-8 (PMC10676424; doi:10.1038/s42003-023-05590-8)
Supplement: Supplementary file 2 — Supplemental Material [file 42003_2023_5590_MOESM2_ESM.pdf]

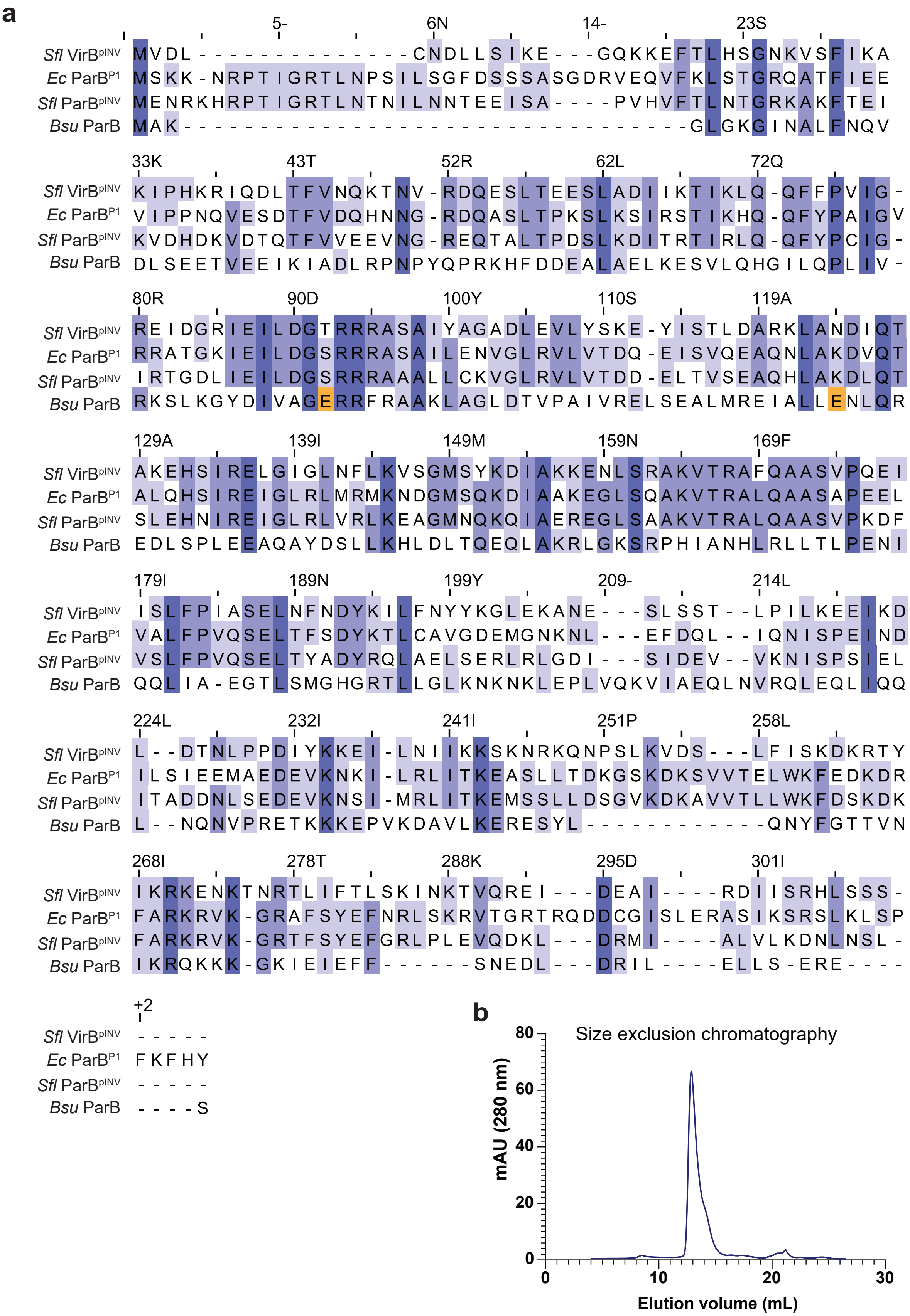

Figure S1: (a) Sequence alignment of full-length Domain organization of *Shigella flexneri (Sfl)* VirB^{pINV}, *Escherichia coli (Ec)* ParB^{P1}, *Sfl* ParB^{pINV}, and *Bacillus subtilis (Bsu)* ParB. The sequence alignment was done on JalView and the residues are colour coded based on percentage identity. **(b)** Size-exclusion chromatography (SEC) profile of purified VirB protein. The SEC profile displays a single peak, indicating a homogenous protein sample.

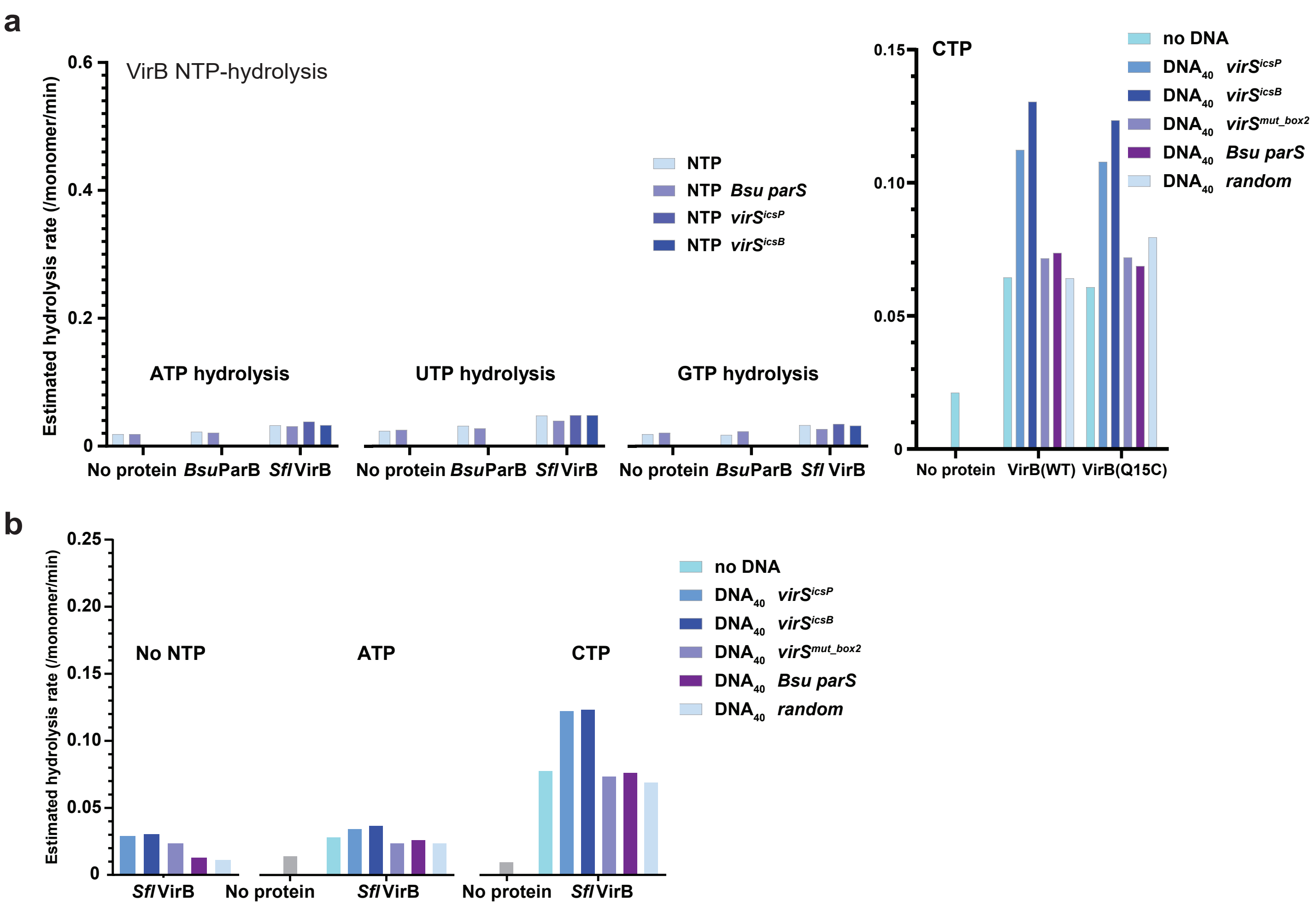

Figure S2: (a) Nucleotide hydrolysis by *Bsu* ParB and *Sfl* VirB(WT) and VirB(C5S, Q15C) measured by malachite green detection of inorganic phosphate. Data for CTP is also shown in Fig. 2B. Additional information can be found in the Materials and Methods section. **(b)** Detection of free inorganic phosphate via Malachite green assay. Same as in Fig. S2A.

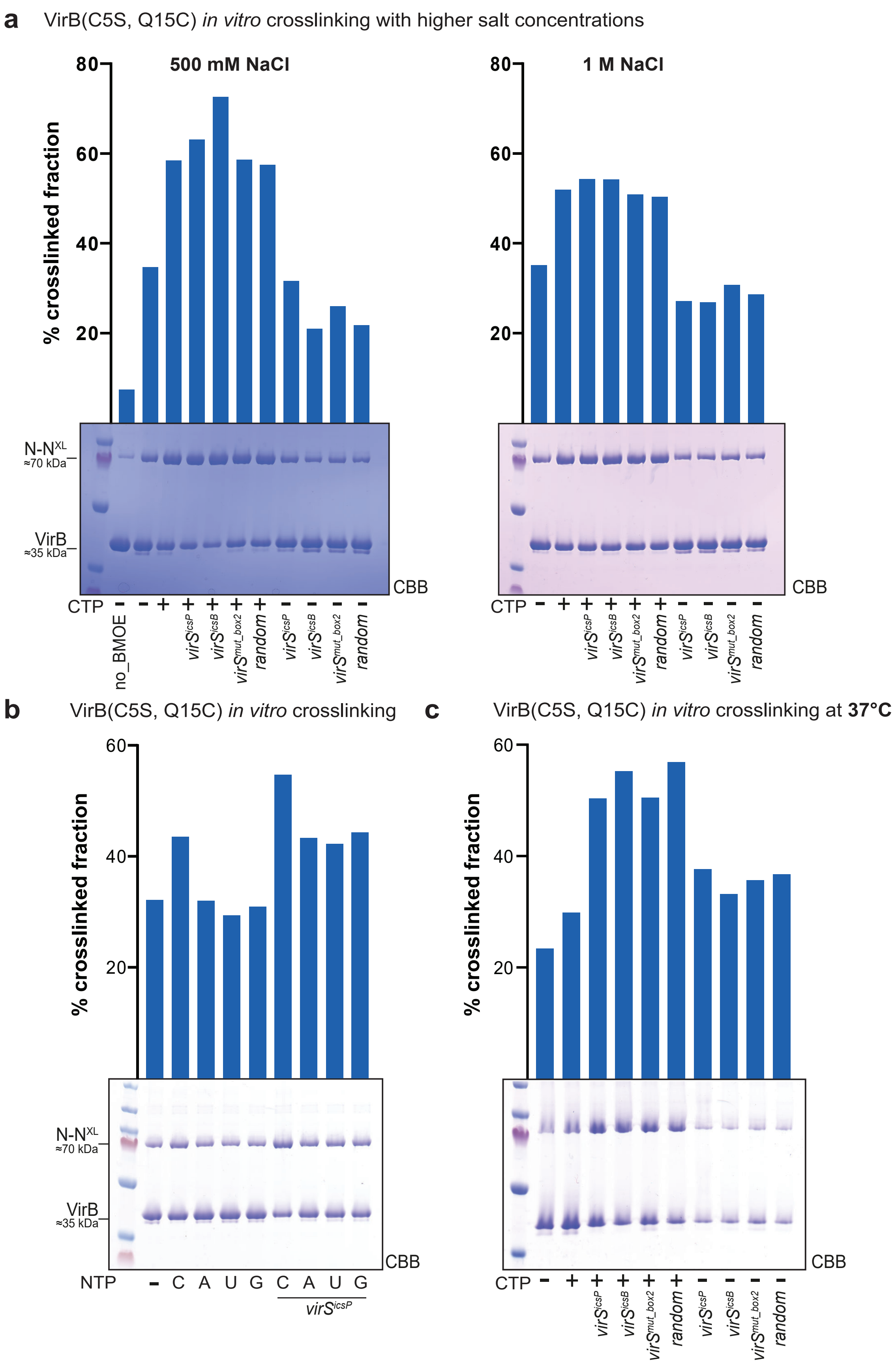

Figure S3: (a) Same as in Fig. 4B: Gel analysis of cross-linking products of purified VirB(C5S, Q15C) in higher salt conditions (500 mM and 1 M NaCl). **(b)** Same as in Fig. 4b: Gel analysis of cross-linking products of purified VirB(C5S, Q15C) in (150 mM NaCl) in the presence of different nucleotide ligands (ATP, CTP, GTP, UTP) with and without DNA. The gel shows no significant VirB N-domain engagement by ATP, GTP, or UTP. **(c)** same as in Fig. 4B but at 37°C. The gel shows comparable cross-linking efficiencies to those observed in Fig. 4B.

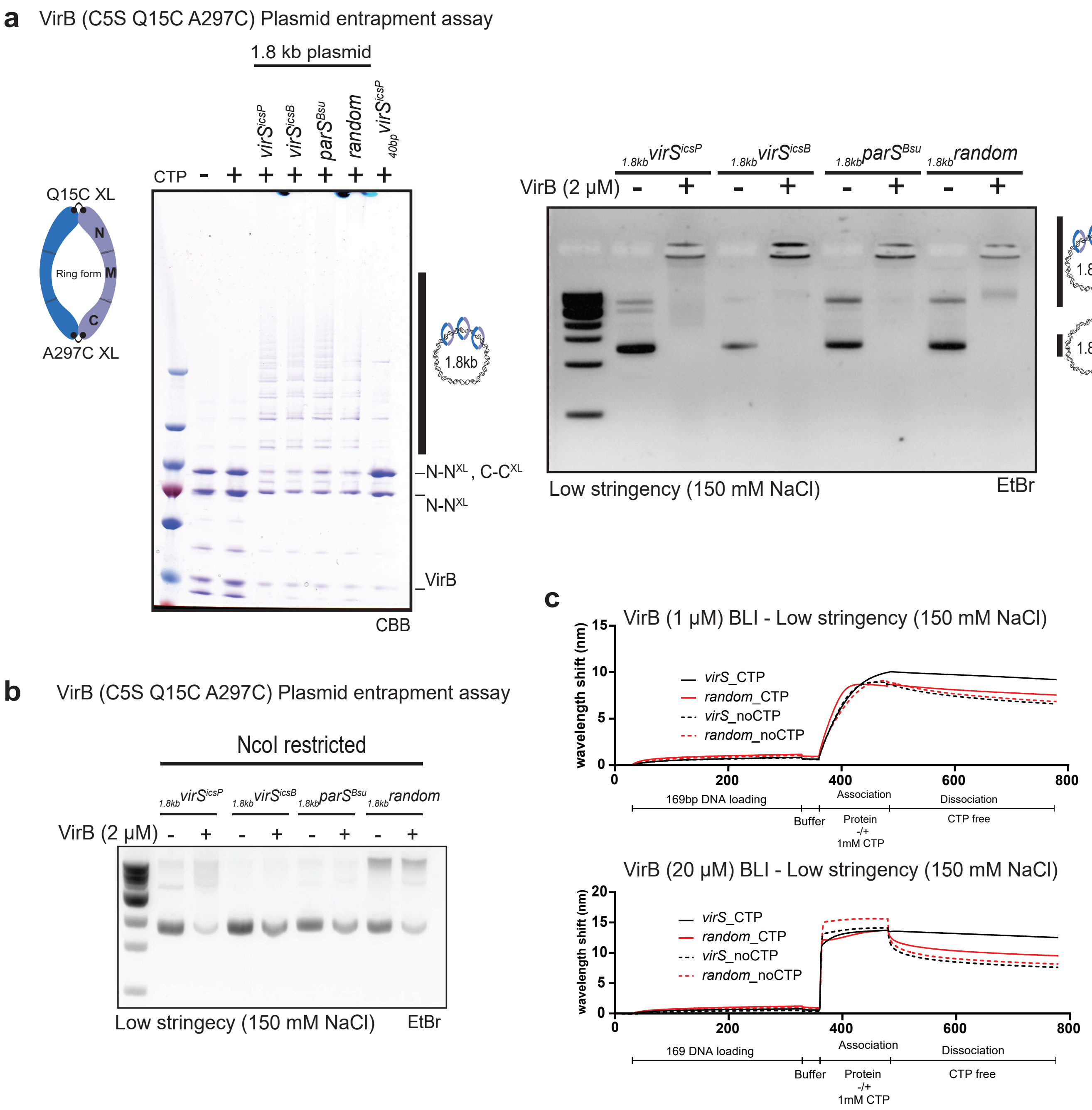

Figure S4: (a) Plasmid entrapment assay by VirB(C5S, Q15C, A297C): polyacrylamide gel electrophoresis analysis of VirB protein species (CBB) and DNA species (ethidium bromide EtBr) from BMOE-cross-linked DNA loading reactions. The buffer used here is low stringency buffer (50 mM Tris-HCl pH 7.5, and 150 mM NaCl). The figure shows the entrapment of circular DNA by VirB, as observed in all four tested plasmids in the presence of CTP. A putative topologically entrapped circular plasmid by double cross-linked VirB dimers (VirB-XX) is marked. **(b)** same as in a with the plasmids being subjected to NcoI restriction (unique cutter) after BMOE-cross-linking. **(c)** Biolayer interferometry (BLI) analysis of VirB (1 and 20 µM) loading onto biotin-immobilized 169-bp *virS* or random DNA. Same as in Fig. 4d but in near-physiological buffer conditions (150 mM NaCl).

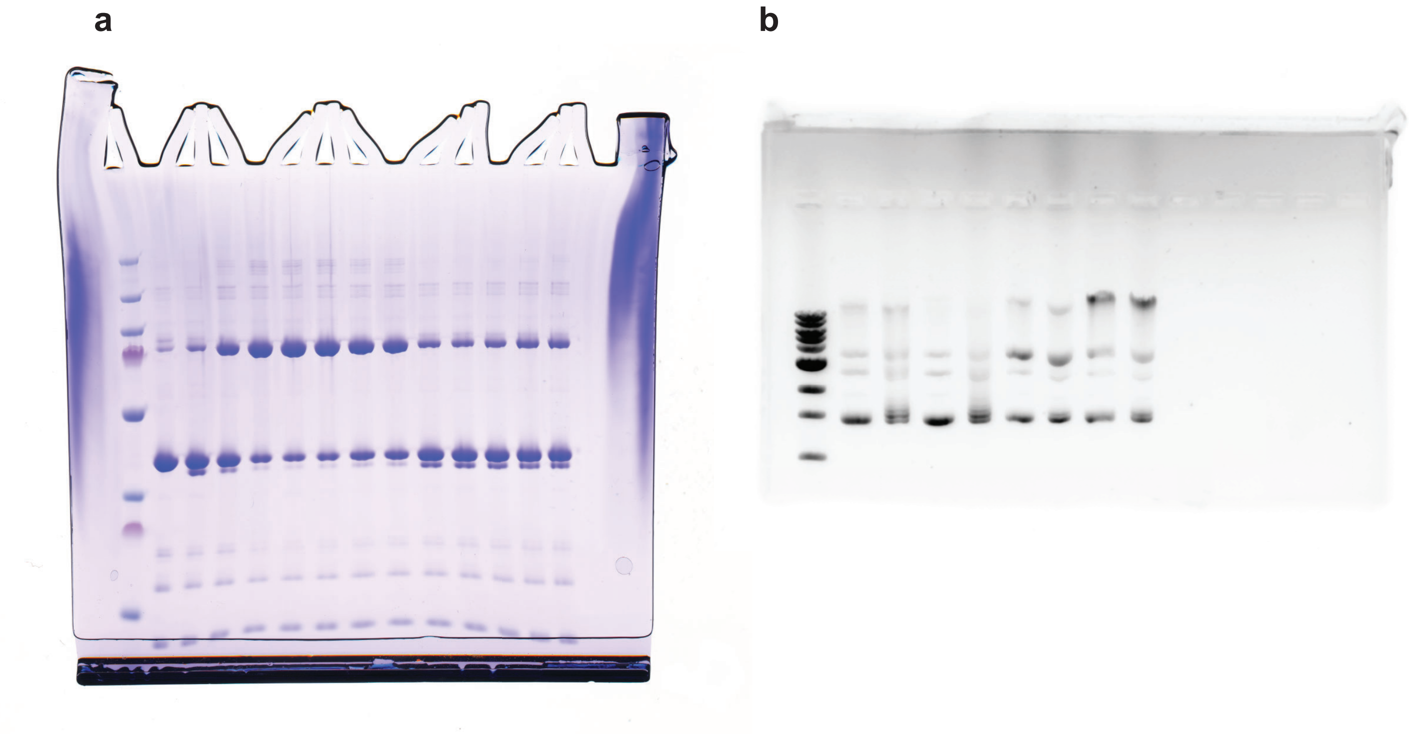

Figure S5: (a) Uncropped and unedited SDS page for Fig. 4b. **(b)** Uncropped and unedited agarose gel for Fig. 4c.

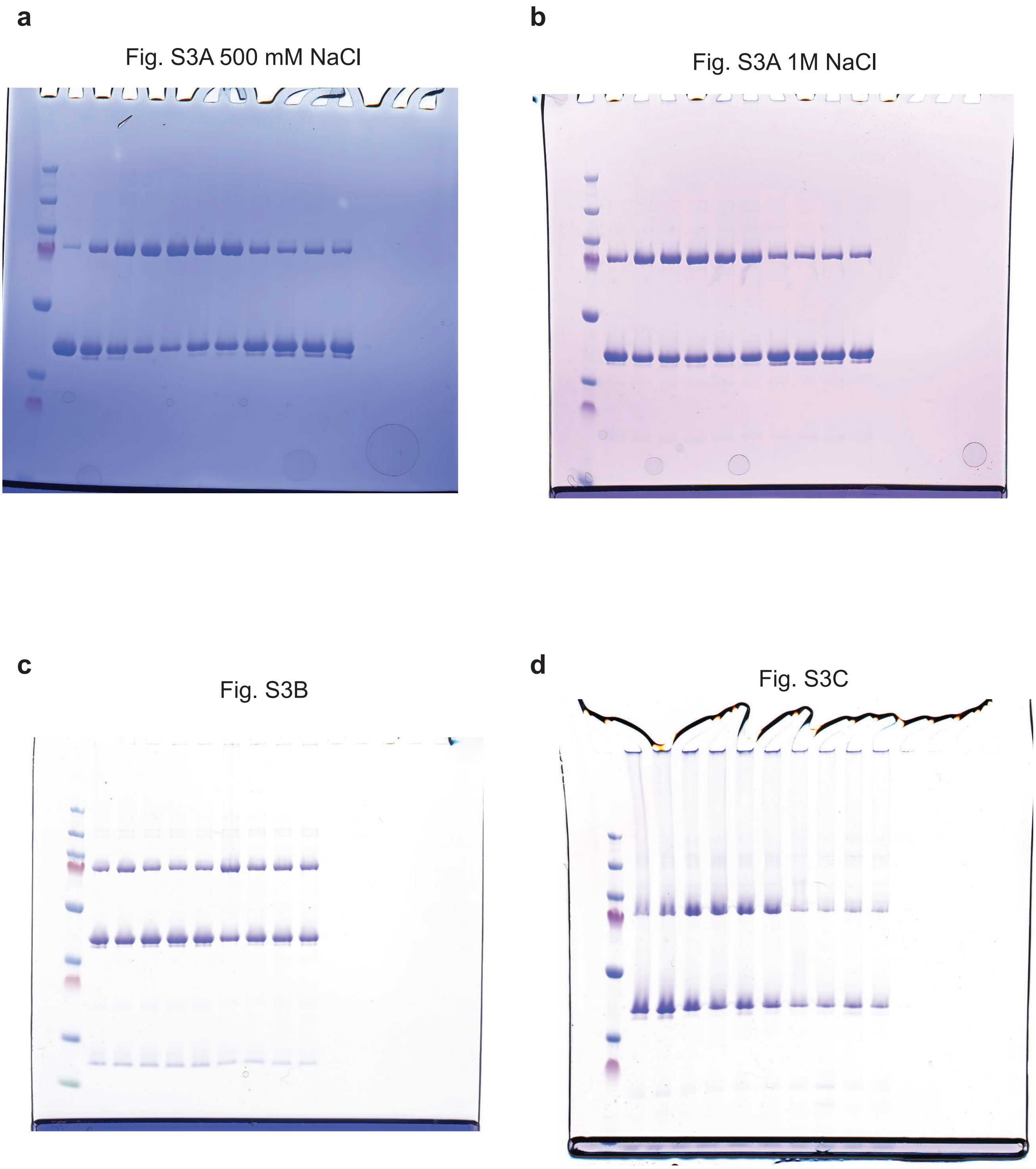

Figure S6: (a,b) Uncropped and unedited SDS page for Fig. S3a 500 mM NaCl and 1M NaCl respectively. **(c,d)** Uncropped and unedited SDS Page for Fig. S3b and S3c respectively.

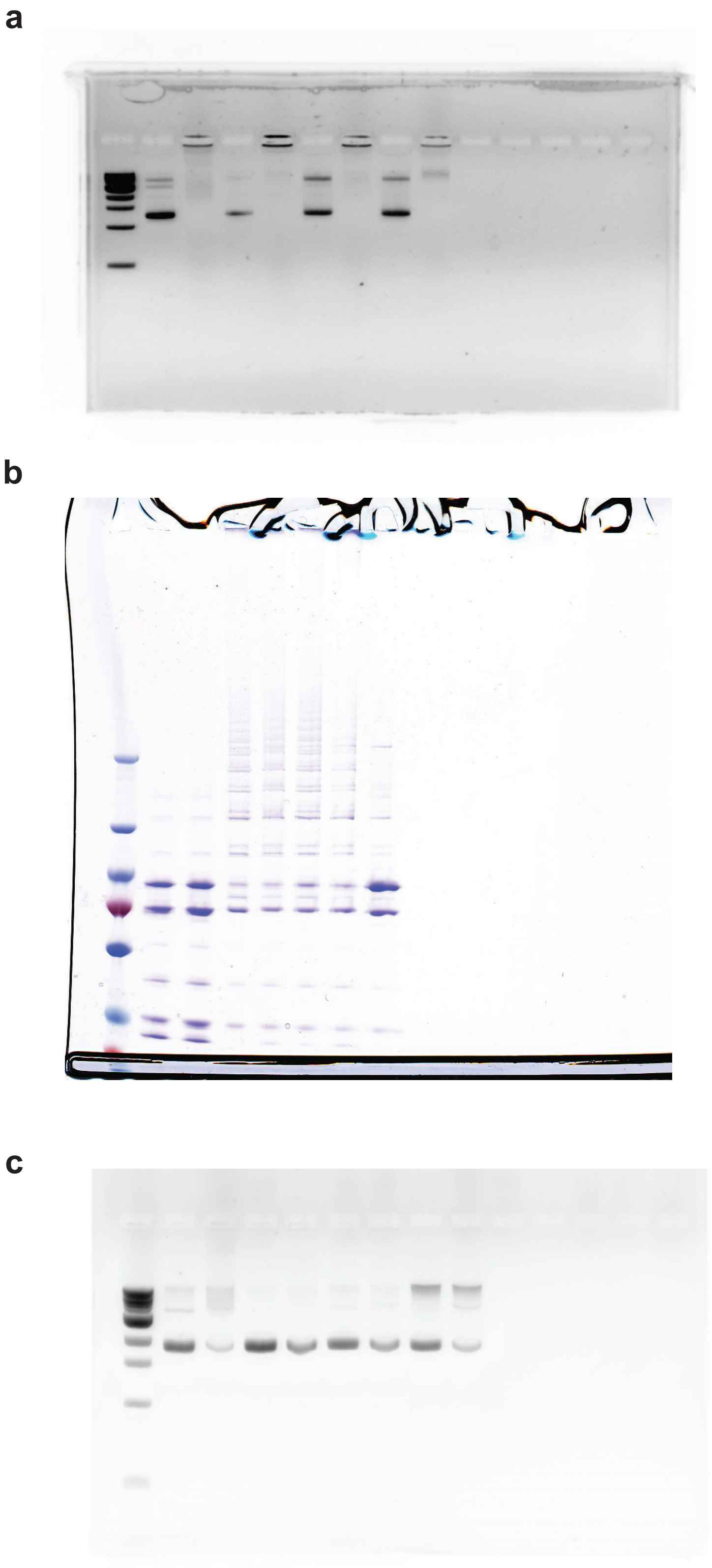

Figure S7: (a) Uncropped and unedited agarose gel for Fig. S4a. **(b)** Uncropped and unedited SDS page for Fig. S4a. **(c)** Uncropped and unedited agarose gel for Fig. S4b.

Supplementary table 1: Primers used in this study.

Identifier	sequence 5'-3'	oligo
**STI706**	ttagttgttctgtttcacgtggaacatctggattacccgc	F DNA_{40} *Bsu parS*
**STI707**	gcgggtaatccagatgttccacgtgaaacagaacaactaa	R DNA_{40} *Bsu parS*
**STN727**	ctccggaatattaggtctcaccatggtggatttgtgcaacga	F for *virB* amplification
**STN728**	ggctcaagcagtgggtctccatccttatgaagacgatagatggcgag	R for *virB* amplification
**STN815**	atacttccggggatttcagtatgaaatgaagtatatttaa	F DNA_{40} *sfl* $virS^{icsp}$
**STN816**	ttaaatatacttcatttcatactgaaatccccggaagtat	R DNA_{40} *sfl* $virS^{icsp}$
**STN817**	tatgtagtgctcgtttcatcatgaaatcccacaagataaa	F DNA_{40} *sfl* $virS^{icsB}$
**STN818**	tttatcttgtgggatttcatgatgaaacgagcactacata	R DNA_{40} *sfl* $virS^{icsB}$
**STO396**	Biotin*cataaacatgcttgataact	F upstream $virS^{icsp}$ to make 169bp DNA fragment (BLI)
**STO397**	Biotin*aggcttggcagtttggataa	R downstream $virS^{icsp}$ to make 169bp DNA fragment (BLI)
**STP048**	ctccggaatattaggtctcaccatggtggatttgAgcaacga	F in *virB* C5S
**STP049**	aataaagtttcctttTGCaaagccaagattcct	F in *virB* I30C
**STP050**	aggaatcttggctttGCAaaaggaaactttatt	R in *virB* I30C
**STP051**	CTCCGGAATATTAGGTCTCAccatggtggatttgAgcaacgacttgt taagtataaaggaaggcTGCaagaaa	F in *virB* C5S Q15C
**STP408**	tatgtagtgctcgtttcatcgctgggccccacaagataaa	F DNA_{40} *sfl* $virS^{mut\_box2}$
**STP409**	tttatcttgtggggcccagcgatgaaacgagcactacata	R DNA_{40} *sfl* $virS^{mut\_box2}$
**STP531**	atacttccgggggcccagctcgacccggaagtatatttaa	F DNA_{40} *random*
**STP532**	ttaaatatacttccgggtcgagctgggcccccggaagtat	R DNA_{40} *random*
**STP553**	ggctcaagcagtgggtctcCatccttatgaagacgatagatggcgagaaa ttatatcccgaataCAttcatctattt	R in *virB* A297C
**STQ245**	attctggatggcactGCGagaagagcatct	F in *virB* R93A
**STQ246**	agatgctcttctCGCagtgccatccagaat	R in *virB* R93A
**STQ247**	ctggatggcactcgtGCGagagcatctgca	F in *virB* R94A
**STQ248**	tgcagatgctctCGCacgagtgccatccag	R in *virB* R94A
